# Supplementary material for: Nematic fluctuations in the cuprate superconductor Bi2Sr2CaCu2O8+δ
Source: Nat Commun. 2019 Nov 15;10:5209. doi: 10.1038/s41467-019-12940-w (PMC6858346; doi:10.1038/s41467-019-12940-w)
Supplement: Supplementary file 1 — Supplementary Information [file 41467_2019_12940_MOESM1_ESM.pdf]

Supplementary Information for "Nematic Fluctuations in the Cuprate Superconductor $Bi_2Sr_2CaCu_2O_{8+\delta}$" by Auvray et al.

Supplementary Figure 1

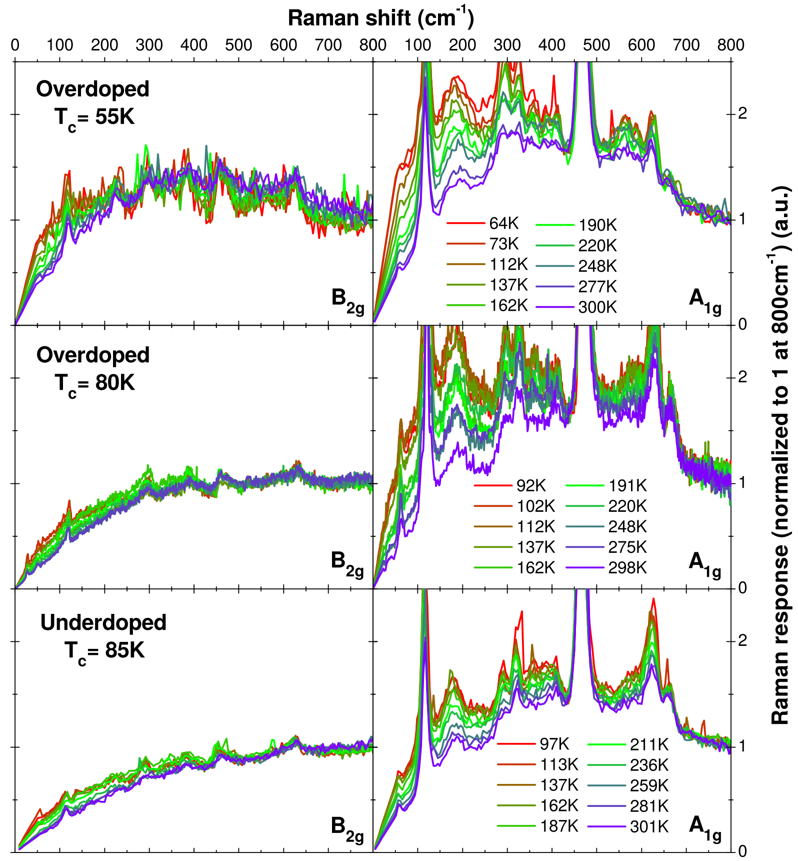

A_{1g} and B_{2g} spectra for the three samples where these geometries were measured. A_{1g} was extracted from $A_{1g} + B_{2g}$ and B_{2g} direct measurements.

Supplementary Figure 2

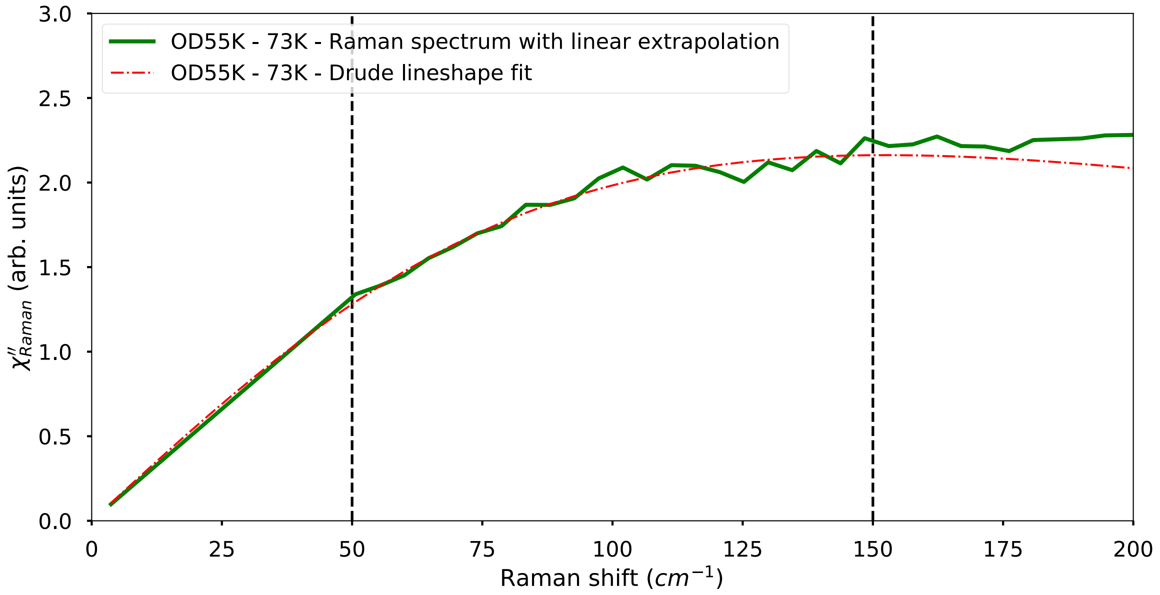

$T = 73\,\mathrm{K}$ spectrum for sample OD55 taken using the 600g/mm grating. Below $50\,\mathrm{cm}^{-1}$, the spectrum is a linear extrapolation of the data acquired above that limit. A Drude lineshape fit is consistent with this bare-bones extrapolation, up to $150\,\mathrm{cm}^{-1}$ above which the Drude model does not properly describe the studied system.

Supplementary Figure 3

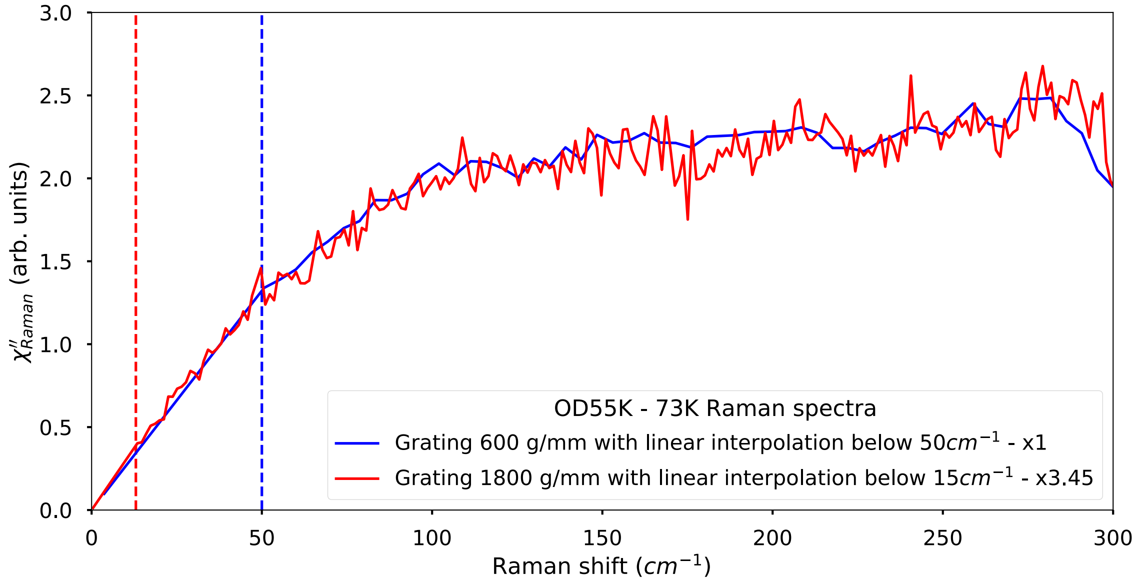

Comparison of Raman spectra of OD55 taken at 73K with 1800g/mm and 600g/mm along with their interpolation at low frequency.

Supplementary Figure 4

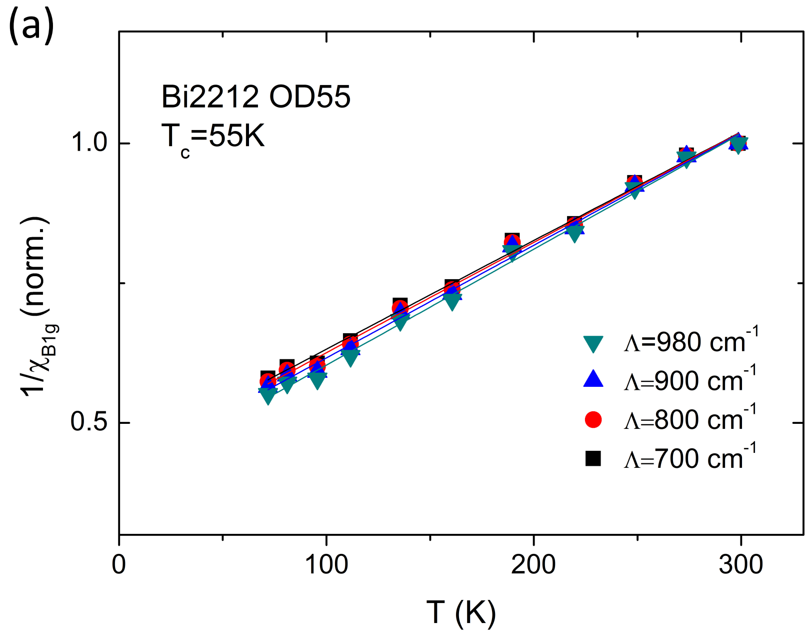

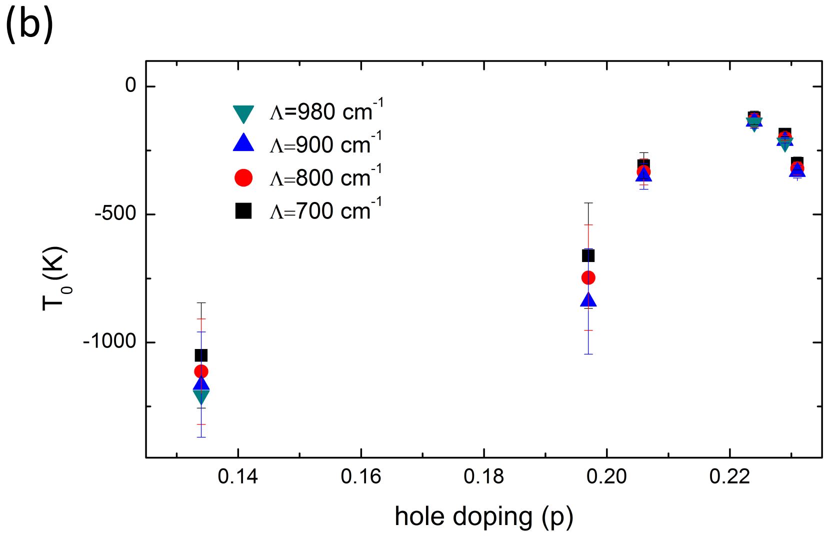

(a) Curie-Weiss analysis of the susceptibility as a function of the cut-off Λ on OD55 crystal. (b) Curie-Weiss temperature T_0 as a function of doping for different Λ. The error bars are the standard error of the Curie-Weiss fits.

Supplementary Note 1

A_{1g} spectra were obtained from $A_{1g} + B_{2g}$ and B_{2g} spectra. The mean intensity ratio between $A_{1g} + B_{2g}$ and B_{2g} raw data was estimated for each series of measurements. The corrected and normalized spectra were multiplied by this ratio, before subtracting the B_{2g} spectra from the $A_{1g} + B_{2g}$ ones.

Supplementary Note 2

Since spectra only span a range of energy starting at $50\,\mathrm{cm}^{-1}$ or $15\,\mathrm{cm}^{-1}$ depending on the configuration used and the quantity we estimate from the spectra is $\chi^0(T) = \int_0^\Lambda d\omega \frac{\chi''(\omega,T)}{\omega}$ it is necessary to extrapolate the values of χ'' down to zero wavenumber. Above T_c and T^*, both a linear prolongation of the spectra down to zero wavenumber and Drude-like fits were performed yielding essentially identical results. For spectra below T_c and T^* a linear interpolation of χ'' was used. For the Drude model the following lineshape was used (see supplementary figures 2 and 3):

$$\chi''_{\text{Drude}}(\omega) = A\frac{\omega\Gamma}{\omega^2 + \Gamma^2} \tag{1}$$

Supplementary Note 3

The cut-off Λ was chosen as the energy scale at which the spectra are temperature independent for all doping. A close inspection of the data reveals that this energy scale is about 800 cm^{-1} for the doping range studied. Integrating further in energy will therefore only add a temperature independent constant to the static susceptibility χ. Since we are only interested in the temperature dependence of the susceptibility. extending further in energy will not affect the T dependent behavior of χ. Furthermore, because the quantity integrated is $\frac{\chi}{\omega}$ and not χ the contribution to the susceptibility coming from the high energy range is small, and only weakly affect the estimation of T_0. This is shown in the supplementary figure 4 where Curie-Weiss fit (ignoring a temperature independent constant as a first approximation) and T_0 values have been extracted using different cut-off Λ ranging from 700 to 980 cm^{-1}. While T_0 values can change by as much as 20%, this remains within the error bar of the fits for most doping. More importantly the overall behavior of T_0 with doping is identical and is therefore robust with the choice of cut-off. The main conclusions of our study are therefore not affected by the choice of cut-off.
